# A focused ultrasound treatment system for moving targets (part I): generic system design and in-silico first-stage evaluation

**DOI:** 10.1186/s40349-017-0098-7

**Published:** 2017-07-24

**Authors:** Michael Schwenke, Jan Strehlow, Daniel Demedts, Sabrina Haase, Diego Barrios Romero, Sven Rothlübbers, Caroline von Dresky, Stephan Zidowitz, Joachim Georgii, Senay Mihcin, Mario Bezzi, Christine Tanner, Giora Sat, Yoav Levy, Jürgen Jenne, Matthias Günther, Andreas Melzer, Tobias Preusser

**Affiliations:** 1 Fraunhofer Institute for Medical Image Computing MEVIS, Am Fallturm 1, Bremen, 28359 Germany; 2 Mediri, Heidelberg, Germany; 3 Institute for Medical Science and Technology, Dundee, Scotland; 4 Universita Degli Studi Di Roma La Sapienza, Rome, Italy; 5 Computer Vision Laboratory, Eidgenössische Technische Hochschule, Zurich, Switzerland; 6 GE Medical Systems Israel, Haifa, Israel; 7 InSightec, Tirat Carmel, Israel; 8 Innovation Center Computer Assisted Surgery, Leipzig, Germany; 9 Jacobs University, Bremen, Germany

## Abstract

**Background:**

Focused ultrasound (FUS) is entering clinical routine as a treatment option. Currently, no clinically available FUS treatment system features automated respiratory motion compensation. The required quality standards make developing such a system challenging.

**Methods:**

A novel FUS treatment system with motion compensation is described, developed with the goal of clinical use. The system comprises a clinically available MR device and FUS transducer system. The controller is very generic and could use any suitable MR or FUS device. MR image sequences (echo planar imaging) are acquired for both motion observation and thermometry. Based on anatomical feature tracking, motion predictions are estimated to compensate for processing delays. FUS control parameters are computed repeatedly and sent to the hardware to steer the focus to the (estimated) target position. All involved calculations produce individually known errors, yet their impact on therapy outcome is unclear. This is solved by defining an intuitive quality measure that compares the achieved temperature to the static scenario, resulting in an overall efficiency with respect to temperature rise. To allow for extensive testing of the system over wide ranges of parameters and algorithmic choices, we replace the actual MR and FUS devices by a virtual system. It emulates the hardware and, using numerical simulations of FUS during motion, predicts the local temperature rise in the tissue resulting from the controls it receives.

**Results:**

With a clinically available monitoring image rate of 6.67 Hz and 20 FUS control updates per second, normal respiratory motion is estimated to be compensable with an estimated efficiency of 80%. This reduces to about 70% for motion scaled by 1.5. Extensive testing (6347 simulated sonications) over wide ranges of parameters shows that the main source of error is the temporal motion prediction. A history-based motion prediction method performs better than a simple linear extrapolator.

**Conclusions:**

The estimated efficiency of the new treatment system is already suited for clinical applications. The simulation-based in-silico testing as a first-stage validation reduces the efforts of real-world testing. Due to the extensible modular design, the described approach might lead to faster translations from research to clinical practice.

## Background

Focused ultrasound (FUS) is increasingly entering clinical routine as an alternative treatment method in different indications [1]. Current clinical FUS applications mostly target static organs. FUS treatment of abdominal targets that move with the respiration of the patient (e.g. the liver) is not yet an available option in clinical routine due to the lack of treatment systems supporting this functionality. The current clinical use cases of FUS for moving abdominal organs include both palliative treatments and patients for whom resection is impossible due to the position of the malignant tissue or co-morbidities [2, 3].

Treating targets in abdominal organs like the liver with FUS is challenging [2]: The target moves with the respiration and may be partially blocked by the ribcage for parts of the respiratory cycle. Real-time temperature monitoring of the treatment needs to be performed using MR thermometry [4]. For moving organs this is even more complicated than for static organs. Thermometry using ultrasound imaging is possible [5], but not yet available for clinical use.

To tackle these challenges, in today’s FUS treatment of moving abdominal targets, the motion of the target needs to be controlled under anesthesia [6–8]: One option is to stop the respiration (apnea) for the duration of active ultrasound application and afterwards let the respiration normalize before starting the next sonication. This procedure, however, results in long treatment duration and the need for general anesthesia. Another approach that is close to clinical application is respiratory-gated sonication [9]. Thereby, the therapeutic ultrasound is active during the respiratory cycle only when the target is detected to be in a certain position. This approach allows for a treatment without general anesthesia, but suffers also from overly long treatment duration due to the reduction of the FUS duty cycle.

In the long run, FUS can become competitive to other thermal ablation techniques as radio-frequency ablation (RFA), microwave ablation (MWA), laser-induced thermotherapy (LITT), and cryoablation. It may even become an alternative to resection and radiosurgery. Whether FUS can be competitive to these well-established techniques strongly depends on results of current and future clinical application of the treatment. In our research we hypothesize that the full potential of FUS in moving organs can be unleashed only with dedicated computer support in planning and control of the treatment.

In this paper we present our efforts from the past years in developing model-based software support for FUS in the moving liver. An integrated system has been developed that shall allow to treat the liver during motion. Here we focus in particular on the system design and on our framework for in-silico testing of the system. We also describe our efforts to simplify the translation of research results to prototypes that may be used in clinical studies.

A major effort in developing a software system for treatment planning and control is quality assurance (QA): *Making sure that the system does what it is supposed to do (efficacy), and, most importantly, that it does no harm (safety).* For safety-critical systems estimates for the proportion of life cycle costs spent on QA and testing go up to 80% [10]. Thus, for industry, the hurdle to develop a FUS treatment system with motion compensation is high because of the risk that FUS may in the end turn out to not be competitive to the above-mentioned established ablation methods.

From the viewpoint of research and development, changing currently available clinical FUS treatment systems to make them capable of motion-compensated FUS is difficult as the clinically approved systems are not open for extensions and are subject to closed specifications. Also, introducing changes to the certified systems means that any validation needs to be re-performed.

For systems of such complexity the effort required for a change in the system grows drastically the later a defect is detected or a new feature should be incorporated (exponential cost-of-change curve [11]).

In our work, we define the requirements and design specification in an open way to simplify extension and collaboration. We aim at reducing the necessary QA efforts by code generation and extensive continuous testing. A virtual system using numerical FUS simulations has been developed and used for first-stage numerical experimental validation. Using this virtual system, automatic execution of system tests is possible and system design parameter studies can be performed to analyze the efficiency of the system. This way, the effort for actual real-world experimentation and validation can be reduced allowing for a more changeable system.

### Contribution

In this article we report on our development of a FUS treatment system including motion compensation with the long-term goal of meeting the requirements for a “Class III Medical Product”. The system allows to perform a single-target motion-compensated sonication during respiratory motion. It is specified and defined in a generic way abstracting from actual hardware components. A first implementation of the system has been performed with clinically available hardware, i.e. an MR device (GE Healthcare Systems, Chicago, USA) and FUS device (Conformal Bone System 2100, InSightec Ltd., Tirat Camel, Israel). To facilitate the translation of FUS research results to the clinic, the treatment system is defined open for extensions and the use with hardware devices of other vendors and may be used as a research platform for FUS experimentation.

A virtual system using numerical FUS simulations is presented and used for first-stage numerical experimental validation. The treatment system interacts with the emulation in the same way it does with real hardware and is provided with realistic data that is influenced by its control commands. A comprehensive numerical FUS simulation during respiratory motion (previously described in detail [12]) is used to predict the treatment effects that would happen in a real FUS experiment. Using this virtual system, automatic execution of system tests is possible and system design parameter studies are performed to analyze the efficiency of the system. One advantage of this approach is that the numerical experiments can easily be observed in the simulation whereas physical measurements in real-world experiments are challenging, time-consuming, and require a lot of resources.

### Related work

**Motion-compensation for FUS.** The challenges for FUS treatment systems imposed by respiratory motion have been addressed by several studies in the literature. Table 1 lists and categorizes the reported approaches. Experimental treatment control systems have been developed by several research groups and validated in ex-vivo and first animal studies. The motion observation approaches include systems that analyze motion surrogates like respiratory belts or use image-based motion tracking of ultrasound or MR images. The FUS control approaches range from gating approaches to real-time beam steering. The systems target both ablation and hyperthermia applications. The validations have been performed case-based as proof-of-concept validations either ex-vivo or additionally on animals (sheep and pigs). While all these approaches show considerable advancement of the methodologies for FUS of moving organs, none provides a prospect to approach any clinical trials with their systems. This is most probably due to system certification impediments.

**Table 1:** Approaches to FUS control systems with motion compensation

Reference (year)	Group/principal investigators	Tracking source	Prediction	FUS control	Validation
Pernot et al. [20] (2004)	Fink, Tanter	3D US-based tracking (using elements of the therapeutic probe), 10-50 Hz	No	Steered (10-50 Hz)	Ex-vivo
Marquet et al. [19] (2011)	Fink, Tanter	3D US-based tracking (using elements of the therapeutic probe), 10 Hz	No	Steered, spiral (10 Hz)	Ex-vivo, in-vivo pig
de Senneville et al. [21] (2007)	Moonen	MR image-based registration (correlation with motion atlas)	2 s delay compensation using pre-treatment analysis of periodic motions (average period Fourier decomposition)	Steered	Ex-vivo (phantom moved by motor)
Ries et al. [22] (2010)	Moonen	2D MR with prospective slice tracking using pencil-beam navigator; 2D optical flow on GPU	3D Kalman-predictor for trajectory anticipation (<114 ms latency compensation)	Steered (>10 Hz)	Ex-vivo, in-vivo pigs
de Senneville et al. [23] (2011)	Moonen	MR (10 Hz), optical flow	Learning motion pattern during preparation	Steered	Hyperthermia, ex-vivo, in-vivo pigs
Quesson et al. [24] (2011)	Moonen	5-slice MR (2.5 Hz)	Not reported	Not reported	In-vivo pig
Holbrook et al. [25] (2014)	Pauly	Respiratory bellow	Look-up table generated in preparation step	32 target presets	Ex-vivo, in-vivo pig
Auboiroux et al. [26] (2012)	Salomir	MR compatible US imaging (≥ 20 Hz), optical flow tracking, 2D	No	Steered (8 Hz), single- and multi-focus	Ex-vivo (ventilator-driven balloon)
Celicanin et al. [27] (2014)	Salomir	MR, 1D pencil-beam navigator (80 ms update)	No	Steered (max 20 Hz)	Ex-vivo, in-vivo sheep

The work of Marquet et al. [19] is the only one addressing both motion compensation and transcostal sonication (using binarized apodization)

**System emulation as a driver for development.** Industry uses emulation of hardware systems that can be employed in the software development process, for example in aerospace software development [13]. Clearly high priority is assigned to safety and reliability in these applications. To allow for a parallel development of hardware and software, in [13], the authors propose simulation-based hardware emulation. Therewith, the hardware and software can be developed iteratively and dependencies are minimized. This also facilitates the application of agile development approaches, in which requirements are not only specified at the beginning of the development but rather evolve during the development and adapt in short cycles. Contrary to the use of numerical simulations for periodic performance assessment (as being done in several industrial applications), in the emulation-based development, simulation is used to provide continuous feedback for collaborative design and development [13].

## Methods

In the following, we first define the generic treatment system that abstracts from actual vendor-specific hardware details. While the system allows the use of different methodological approaches to solve FUS-related tasks (thermometry, focusing), it abstracts also from any implementation details of these. After the definition of the generic treatment system, we provide details for our implementation of the system with a clinically available MR device (GE Healthcare Systems, Chicago, USA) and a FUS device (Conformal Bone System 2100, InSightec Ltd., Tirat Camel, Israel). Closing the section, the numerical-simulation-based approach for a first-stage validation of the system is presented.

### Specification and quality assurance approach

A thorough requirements analysis involving a multi-disciplinary team of radiologists and engineers was performed. According to these stated requirements, a detailed design specification of a generic system was defined. The details of these specifications go beyond this paper. We only briefly describe the main components and concepts of the system. The main hardware devices of the system are the MR imaging device and the therapeutic ultrasound transducer. Both devices have a software representation within the system controlling the devices by using their programming interfaces. The most important software components of the system include the motion compensation system and the temperature calculation component.

#### MR imaging device

The generic specification states that an MR device shall be able to acquire a breath-hold planning image. It does not specify a certain MR sequence. Furthermore, it shall allow the acquisition of a transducer calibration image that can be used by the system to identify the spatial orientation of the FUS transducer with respect to the patient. Most importantly, the MR device shall allow to acquire monitoring images with a high update rate and as little delay as possible. The treatment controller system derives motion observation and temperature information from these images. To simplify the system, we have restricted our developments to MR imaging although, generally, the system could use multiple sources of tracking data (respiratory belt, US tracking, MR tracking) simultaneously. The main intention for our restriction is to avoid the introduction of another hardware system like an MR-compatible diagnostic imaging device in the current development stage. For future versions, we expect a benefit from combining US tracking with MR tracking. We do not plan to use external tracking (like respiratory belts) as external motion is not necessarily correlated with the liver motion [14].

#### Ultrasound transducer system

One of the key requirements of the transducer system is its capability to change the focus very fast. Two kinds of ultrasound transducer control approaches can be handled by the generic treatment system:

1. Transducer systems with a real-time interface (fast enough to be controlled with a full parameter set of phases and amplitudes for all individual elements in real time);
2. Hardware that works with preset configurations, which need to be uploaded before the actual sonication and that can be activated sufficiently fast during sonication.

Transducers that provide a real-time interface are easier to handle, since a preset-based interface requires pre-computing a suitable set of presets based on an observed motion trajectory. In our system, in case of a multi-baseline thermometry approach, the presets can be determined from the motion information in the baseline image collection. This assumes that the motion states in the baseline collection are representative for the duration of the sonication. Focusing of the ultrasound beam is computed within the system using a ray-based method [12]. For complex focusing approaches that are not real-time capable, a preset-based approach can be used also with transducer systems with a full real-time API. The treatment system provides the focusing component with deformed anatomical structure maps to allow for trans-costal focusing approaches.

#### Motion compensation system

For motion compensation, the following steps are repeatedly performed during monitoring of the therapy. The computational pipeline starts with the image acquisition: image reconstruction, image send over network, receive and image assemble on the treatment controller. Based on each newly received image, the controller performs a motion analysis: Feature tracking is used to identify local motion of anatomical structures. The features are then related to the reference respiratory state of the planning image to allow for a mapping of planning data to the current motion state. To steer the ultrasound focus to the current target position, a temporal prediction needs to be performed compensating any delays (the delay from image acquisition to the analyzed motion state, the delays introduced by focusing, sending the FUS control parameters to the hardware, and the hardware delay of the FUS device).

**Fig. 1:**
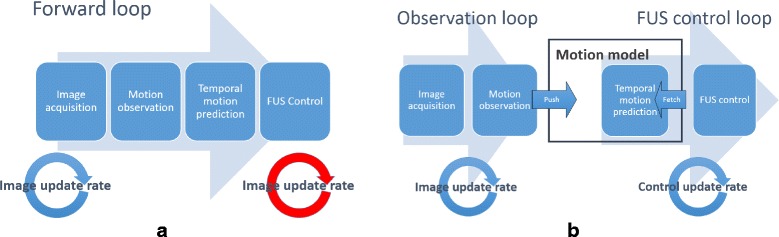
Motion compensation: Decoupling the FUS control from the image update rate. The forward loop **a**) restricts the FUS control loop to run with the same rate as the monitoring imaging. To decouple both loops, in **b**), a motion model is introduced allowing for a flexible choice of control update rate

Instead of implementing all this in a feed-forward loop, we propose to decouple the observation part of the loop from the control part. Figure 1 a illustrates the problems of a feed-forward approach: The FUS system is controlled with the same update rate as the imaging rate. As a remedy, a motion model is introduced (see Fig. 1 b) to break the forward loop into two concurrently executing loops: i) The motion observation loop starts with each new monitoring image and performs the tracking, relates the data to the reference motion state and feeds the new motion information into the motion model to update its state. ii) The FUS control loop simultaneously uses the temporal prediction functionality available in the motion model to compute FUS control parameters for a predicted motion state and upload the parameter set to the FUS hardware.

Thereby, the observation and control loops can be executed with different update rates and can be adapted to the actual constraints of the hardware devices with great flexibility. This comes at the cost of slightly higher overall system latency that needs to be compensated by the motion model.

#### Temperature calculation component (Thermometry)

The specified interface of the temperature calculation component would allow both multi-baseline and reference-less proton-resonance frequency shift MR thermometry approaches. Currently only the multi-baseline approach is implemented. The temperature calculation component is queried by the treatment system core whether it requires baseline images. Accordingly, the system acquires as many baseline images during respiratory motion as the component requested. Then, in case of a multi-baseline thermometry approach, for each new monitoring image the most similar baseline image is determined based on the magnitude images. Consequently, the temperature is computed using the phase information of the current and baseline image. The resulting temperature image is mapped to the reference respiratory state and can be easily overlaid on the planning image to allow for a static-like monitoring of the therapy. This is intended to facilitate the visual inspection by the physician. For the reference-less thermometry, the only difference is that no baseline collection is acquired and for each new monitoring image, the baseline phase image would need to be estimated from the current image itself. A combination of both approaches would also be possible and would be an implementation detail of the temperature calculation component.

### FUS system implementation

**Fig. 2:**
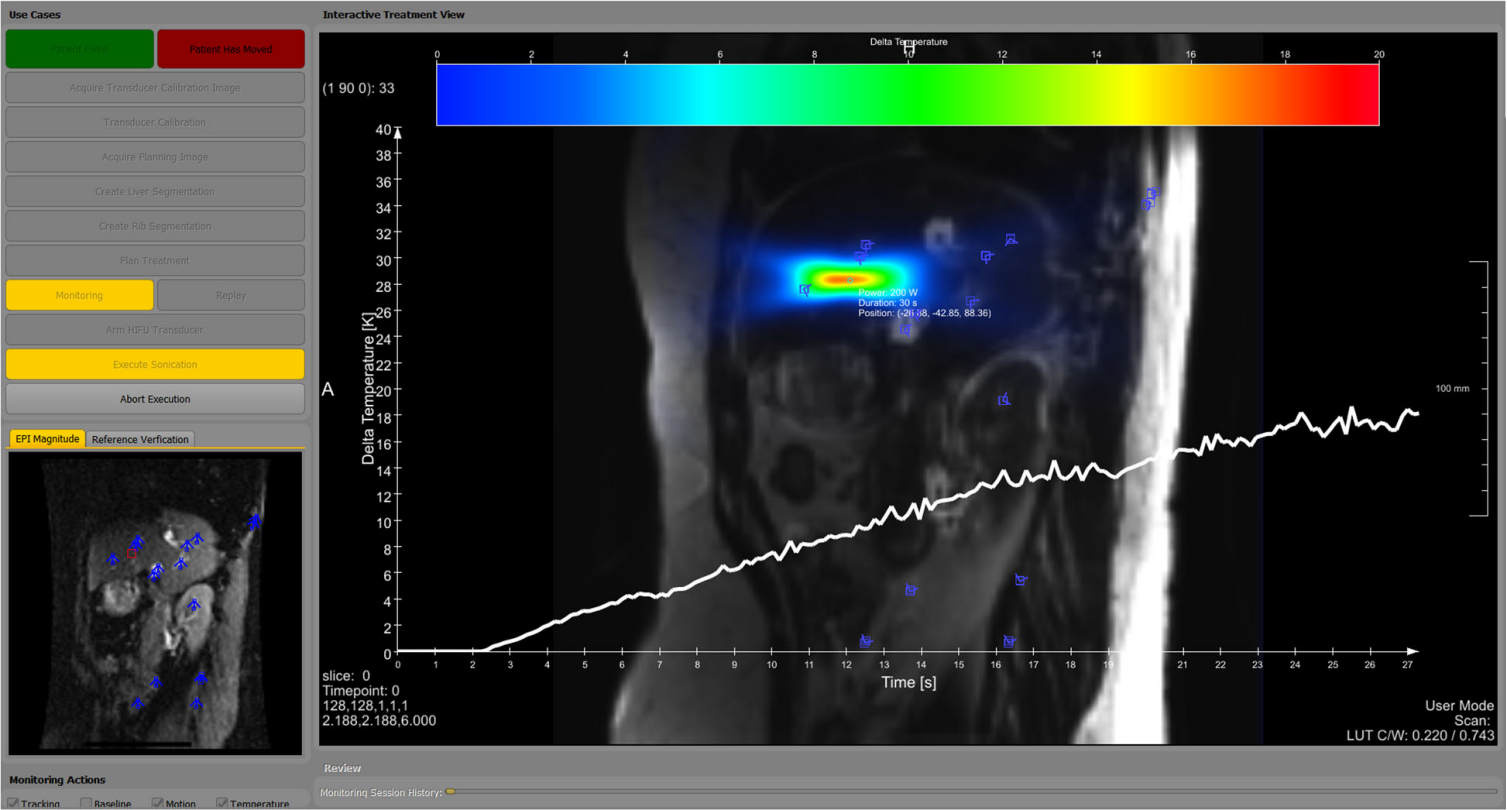
Treatment system user interface with test data

We have implemented a fully functional system according to the above-mentioned specification. Figure 2 shows the graphical user interface during execution of a sonication in emulation mode. The system is integrated with MR devices from GE (GE Healthcare Systems, Chicago, USA) and the Conformal Bone System 2100 (CBS) (InSightec Ltd., Tirat Camel, Israel) FUS device. Echo planar imging (EPI) is used for fast monitoring and the proprietary GE API is used to receive the image data after reconstruction on the scanner side. The monitoring images are used for both motion observation and temperature monitoring. The current system consists of a multi-baseline thermometry component and a preset-based transducer controller. Details on the actual system implementation and hardware integration will be reported in a following publication.

### Emulating the hardware system and modeling the patient and therapy

For a first-stage validation of the system, we have replaced the actual hardware by a virtual system. The hardware and the effects of the treatment to the patient are emulated and numerically simulated. To simulate the resulting temperature in the patient’s body, we incorporated a previously described numerical simulation method for FUS during respiratory motion [12]. The key advantages of using simulation in the process of system development are flexibility, responsive feedback, automatic execution of tests, and the possibility of checking the system efficiency for a wide range of hardware system characteristics like update rates and motion patterns. A detailed view into the inner state of the numerical experiment allows for a better assessment of the effectiveness of the system. Automated testing and evaluation of new components and its influences are possible through this. Elaborate and time-consuming real-world experiments still need to follow, but only after the emulation-based numerical experiments have shown the effectiveness of the system.

The hardware emulation replaces the two main hardware devices involved: The MR imaging device and the FUS device. The treatment system interacts with the emulated system as with the real hardware unaware of which execution system is actually connected. The emulation needs to produce realistic and meaningful data for the treatment system, e.g. monitoring images showing organ motion in real time. To this end, GPU-based image deformation is used to generate the required monitoring image data. The underlying respiratory motion pattern is replayed from actual recorded patient data. We then use our numerical FUS simulation during respiratory motion to simulate the patient status and to generate the image data for the application. This involves simulating ultrasound propagation and heat diffusion during the deformation and motion of the organs. For details on the numerical method and our verification and validation tests thereof the reader is referred to [12]. For the present studies, the FUS simulation does not need to be performed in real time as the treatment system does not analyze temperature feedback yet. Thus it is done following the execution of a sonication to minimize performance side effects on the real-time treatment system. To test the application we let the following emulation parameters vary:

- monitoring image duration (the rate at which monitoring images are produced);
- imaging delay (delay between acquisition and availability of a monitoring image);
- ultrasound shot duration for which the focus is kept static (the motion trajectory is broken down into multiple discrete short static foci called *shots*);
- the transducer type (preset-based or real-time control).

### Temporal motion predictors

A motion predictor uses the latest tracking information to predict the target position for a requested time in the future. We here compare two prediction approaches:

- a linear-extrapolation-based predictor uses the last two tracking samples and extrapolates by a linear fit into the future;
- a history-based predictor that (assuming periodic motion) uses the latest tracking samples to find the most similar motion state in the history of motion states. The prediction of the target position thereby is performed by an interpolation in the historical data (the history continuously grows with new motion samples).

**Fig. 3:**
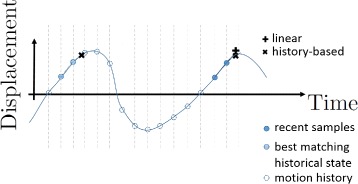
Example of the temporal motion prediction approaches: The linear-extrapolation-based predictor (**+**) overshoots at the turning point of motion. The history-based predictor (**x**) better handles this case by finding the best match in the history of samples and uses the historical state for the prediction

Figure 3 illustrates the history-based prediction approach. Another motion model that we have investigated which is not yet part of the evaluation is described in [15].

### Validation

As mentioned above, quality assurance (QA) is the main effort in translation from research demonstrators to clinical prototypes that may be used in studies. From the two major aspects of *efficacy* and *safety* that have to be shown by QA and validation we focus on the efficacy in the following. Further results on experimental validation have been reported in [16].

We have designed a suite of test scenarios that is based on the emulated system in order to analyze the performance of the treatment system. To quantify the performance of the system, i.e. its efficacy, we aim at comparing it with static sonications. Thus, we determine an efficiency factor for a certain choice of component implementations and system parameter values. This efficiency factor quantifies how much energy is delivered to a moving target in contrast to a static target, see below.

#### Numerical study

In our in-silico study the system parameters are varied over the following ranges:

- monitoring image duration (100 ms, 200 ms, 300 ms);
- ultrasound shot duration (50 ms, 100 ms, 200 ms, 300 ms);
- monitoring image delay is fixed to 150 ms;

Furthermore, we test different choices for motion tracking, motion prediction, and emulated transducer types:

- a Bayesian tracking [17];
- the linear-extrapolation-based and the history-based motion predictors described above;
- emulated real-time and preset-based transducers with varying number of available preset configurations.

To identify the efficiency limit of a system constrained to the above system parameter values, we need to use ground-truth tracking and prediction components. The ground-truth components fulfill the software interface definitions of the tracking and the prediction components and they internally know the underlying test motion at all times. Using these test components the system is provided with exact motion information.

#### Ground-truth respiratory motion and generation of test data

**Fig. 4:**
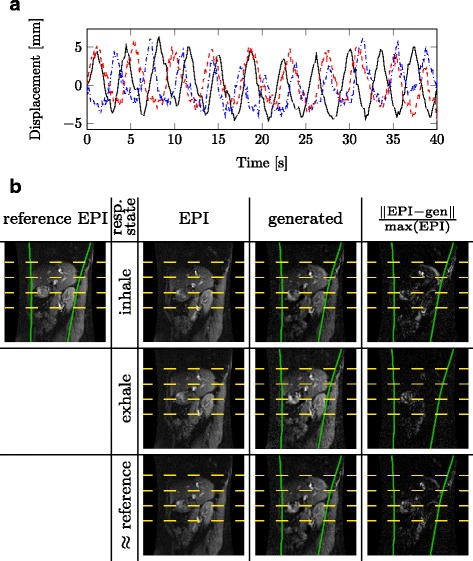
Generation of test-data: The plot in **a**) shows the motion patterns of tracked liver features derived from EPI image sequences of three different volunteers. For our studies, we use the black-solid motion pattern to generate monitoring images. The images in **b**) show data for different motion states comparing the real EPI images and the generated data for the same respiratory state. The *dashed yellow lines* are given to facilitate comparison. The *green solid contours* show the manually delineated sliding boundary between inner organs and ribcage. The *last column* shows images highlighting the differences between the EPI and the generated image, normalized with respect to the range of the EPI image

To generate suitable and controllable test data for respiratory motion we acquired EPI image sequences of volunteers [15]. Respiratory motion patterns are derived from the EPI images for a feature point in the liver. Figure 4 a shows 40 s of the acquired respiratory motion patterns for some feature points in the liver. The motion patterns *p*(*t*)∈[*p*_min_,*p*_max_] are then used as a basis for generating image displacements. Additionally, a scale parameter *s* is introduced, to scale the motion pattern. The displacement function **d**(*t*)∈*I* *R* ^3^ at time *t* is

1 $$ \mathbf{d}(t) = s\,p(t)\,\mathbf{v},  $$

where **v**∈*I* *R* ^3^ is the motion direction unit vector (superior-inferior direction). Using the scale parameter *s*≥0, the motion can be reduced to the static case (*s*=0) or increased to simulate exaggerated motion (*s*>1).

To generate an EPI magnitude image at some time *t*, we first find the EPI magnitude image that is most similar to the planning image respiratory state. Figure 4 b shows this image in the leftmost column.

We then add noise to this image as described below. Using Eq. (1) we translate the noisy image to the moving state. Note that in fact we use just a single translation vector **d**(*t*) here. Thus no deformation of the liver is modeled. A manual marking of the abdominal wall is used to keep the ribcage static by applying the displacements only to the inner organs that slide along the abdominal wall. Figure 4 b visualizes the generated test data for different respiratory states.

To be able to introduce noise we build a noise model based on the EPI images. To this end, we register 50 images of the EPI sequence to the reference EPI magnitude image. Registration was only applied to the inner organs and was based on a translation model. The differences between the registered images and the reference image were visually inspected to ensure that they contained negligible motion artifacts in the liver. During data generation, these difference images were then sequentially added to the reference EPI image to simulate appearance changes. Compared to directly using the original sequence with the registration results, this approach has the advantage of full control over the motion (especially image rate and furthermore magnitude to simulate exaggerated motion). Changing the image rate in a replay of the original sequence would also change the speed of motion, which is undesired.

#### System efficiency factor

The peak temperature rise *d* *T*_static_ at the target resulting from a 10 s single-focus application of 200 W ultrasound energy in a non-moving domain will be used as the reference for establishing an efficiency factor for the FUS system validation in the moving case. Clearly, the temperature rise *d* *T*_moving_ during motion will be less or equal to *d* *T*_static_. Based on this, we define the efficiency of the moving system for a specific motion case to be

2 $$ \eta = \frac{dT_{\text{moving}}}{dT_{\text{static}}}, \quad 0\% \leq \eta \leq 100\%.  $$

For example, if the system has an efficiency of 80*%* for a certain motion pattern, the resulting temperature rise at the target during motion is equivalent to 80*%* of the temperature rise introduced in the static case. The value of *η* can also be interpreted as an energy efficiency: A 100 W sonication in the moving case results in the same peak temperature rise as an 80 W sonication in the static case.

We use the peak temperature rise at the planned target position as the metric for our studies as it gives good results only if the focus in the moving case is both as sharp as in the static case and also at the correct location. Assessing an average over a region around the target might underestimate an unintended focus dislocation. The main effects influencing the peak temperature rise at the planned target are firstly the quality of motion compensation (how accurately was the actual target motion followed by the moving focus) and secondly the heat diffusion process (how much heat is conducted from the heated target to the surrounding tissue). The second effect is largely influenced by the choice of tissue parameters. By using a relative metric that compares static and moving case for the exact same tissue parameters, however, we eliminate this dependency to a large extent.

## Results

### Maximum efficiency during motion

**Fig. 5:**
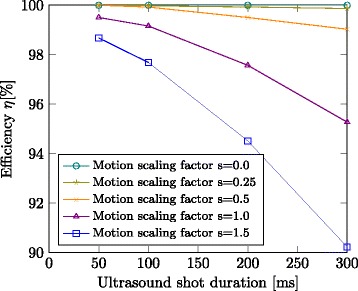
Maximum efficiency for motion compensated FUS for different choices of ultrasound shot duration and different motion scale factors

**Fig. 6:**
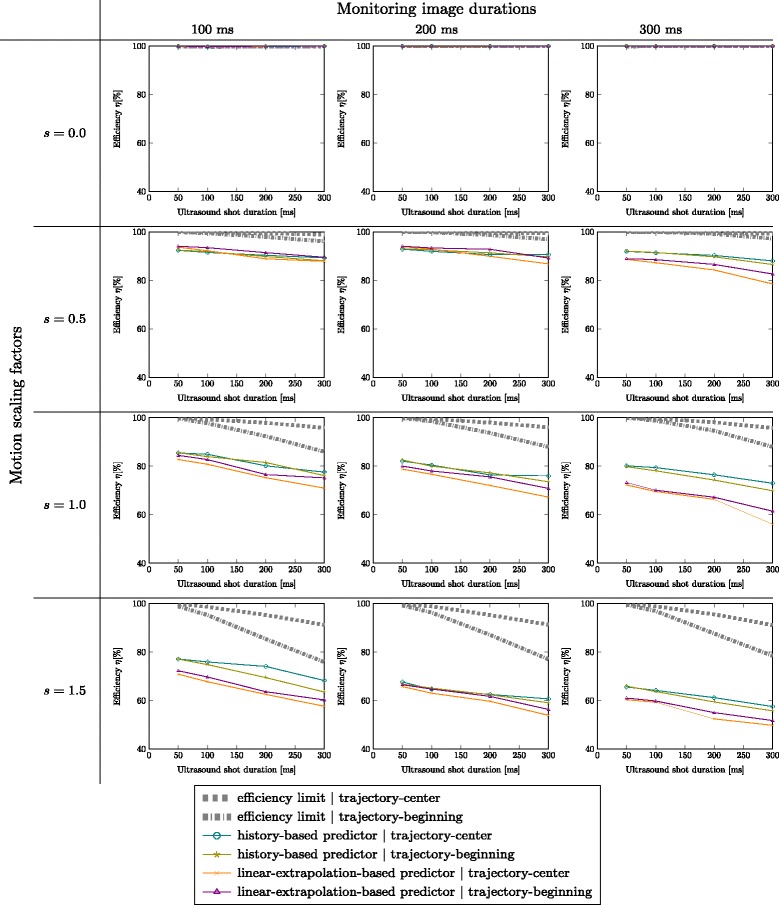
Efficiency evaluation of the system using temporal motion predictors in combination with the ground-truth tracking data. The *rows* represent different motion scaling factors while the *columns* represent different monitoring image duration (inverse of image rate)

For a certain choice of ultrasound shot duration and specific type of motion, the maximum efficiency that can be reached is identified using the test-ground-truth tracking and prediction components together with an emulated real-time ultrasound transducer. Figure 5 shows the efficiency plots over ultrasound shot duration for different motion scaling. These results give the efficiency limits of the treatment system if provided with perfect motion predictions. For subtle motion (*s*=0.5), even with a long ultrasound shot duration of 300 ms, the system efficiency can reach *η*=99*%* and can increase to almost 100*%* for decreasing the ultrasound shot duration to 50 ms. In case of normal respiratory motion (*s*=1.0), the efficiency can be better than 95*%* for 300 ms shot duration and can increase to above 99*%* for 50 ms shot duration. Exaggerated motion (*s*=1.5) might be compensated with an efficiency of 90*%* in case of 300 ms shot duration and can increase to about 99*%* for 50 ms shot duration. By design, the ground-truth motion prediction is invariant to the imaging update rate which can be seen in Fig. 6 below (similar efficiency limits across columns).

### Efficiency of temporal motion predictors

We here evaluate the system efficiency when equipped with different motion predictors. The reference again is the achieved temperature rise in the sonication with no motion. Tested motion predictors are the linear-extrapolation-based and the history-based predictor. As stated before, the ultrasound focus is kept static for the shot duration. During this shot, the target moves continuously along a trajectory.

Since we have de-coupled the observation loop from the control loop (cf. Fig. 1) we may purely concentrate on the small time window of the shot duration. For the focusing we can choose any point on the predicted trajectory within this time window to achive the short-time static sonication. However, we also need to take into account that the error of the prediction increases with prediction time.

Theoretically, shooting at the trajectory point in the middle of the shot duration should best approximate the trajectory. But this shooting at the center of the trajectory also increases the prediction time by half of the shot duration. To assess this effect, we evaluate both prediction methods for targeting at the predicted trajectory at i) the beginning and ii) the center of the shot duration time window.

To separate from tracking errors, the test-ground-truth tracking is used providing accurate tracking data to the motion predictors. All experiments are performed using an emulated real-time transducer. We thus can evaluate the error introduced by the different predictors. Figure 6 shows the efficiency plots over ultrasound shot duration for different motion amplitude factors (0.0, 0.5, 1.0, 1.5) for different monitoring image durations (100 ms, 200 ms, 300 ms; columns).

The first row shows the efficiency for all motion predictors for the static case (*s*=0.0) to verify that all of them can handle the static case. The other rows show the efficiency over shot duration for scaling factors of *s*=0.5, *s*=1.0, and *s*=1.5. We see that all motion predictors can handle the static case and have near-perfect efficiency. In all cases, the efficiency limit for targeting the trajectory center (dashed limit line) is higher than for targeting the beginning of the trajectory. This, however, represents the limit that is computed using the ground-truth predictor that provides the system with error-free motion predictions. When analyzing the imperfect predictors, firstly it is evident that in almost all numerical experiments, the history-based motion prediction performs better than or equally well as the linear-extrapolation-based predictor. Only in the case of 100 ms image duration and reduced motion (*s*=0.5), the linear prediction is slightly better. The plots furthermore show decreasing efficiency for increasing monitoring imaging duration (meaning decreasing image update rate). Sources of this error are the greater temporal prediction horizon and the associated greater prediction error, and also the inferior temporal sampling of the motion. The history-based predictor shows better efficiency and less dependency on image update rate than the linear-extrapolation-based predictor. This meets the expectations as the history-based predictor can better handle the inversion of motion directions at the turning points (see also Fig. 3). The linear-extrapolation-based predictor cannot handle the turning points and thus shows greater dependency on image update rate. We conclude that the history-based predictor is better suited for the available image update rates. Regarding the choice of targeting the beginning or the center of the motion trajectory during a shot with fixed focus, we do not see a clear improvement of shooting at the center. The better approximation of the trajectory on the one hand and the increasing prediction error on the other hand cancel each other out. As targeting the center should be theoretically better and in our tests it does not show worse efficiency than targeting the beginning of the shot, we favor shooting at the center of the trajectory.

### Efficiency of motion tracker

The efficiency of the Bayesian motion tracking is evaluated in the combination with the temporal motion predictors. All tests are performed using the emulated real-time transducer. We compute the efficiency loss *η*_*Δ*_ associated with the Bayesian motion tracking by subtracting the efficiency when using the Bayesian tracking (*η*_Bayesian-tracking_) from the efficiency when using the test-ground-truth tracking (*η*_ground-truth_), with both employing the same temporal prediction:

3 $$ \eta_{\Delta} = \eta_{\text{ground-truth}}-\eta_{\text{Bayesian-tracking}}.  $$

Table 2 lists the results of this investigation. The mean efficiency loss *η*_*Δ*_ over 120 simulated experiments with varying image durations and ultrasound shot durations is 4.9% with a 95%ile loss of 10.8%. In combination with the history-based motion predictor, the efficiency loss is far less than in combination with the linear-extrapolation-based motion predictor.

**Table 2:** Efficiency loss associated with the Bayesian tracking in combination with the motion predictors

Motion predictor	Mean *η*_*Δ*_	95%ile *η*_*Δ*_
Linear-extrapolation	6.4 %	12.9 %
History-based	3.3 %	7.9 %
Overall	4.9 %	10.8 %

### Efficiency of preset-based ultrasound transducers

**Fig. 7:**
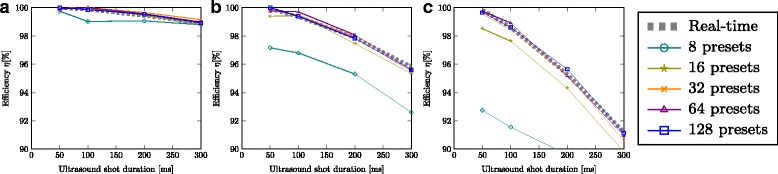
Efficiency evaluation of preset-based transducers against real-time transducers for respiratory test motion with scale factor **a** *s*=0.5, **b** *s*=1.0, **c** *s*=1.5

To assess the error that is introduced by using a preset-based transducer instead of a real-time transducer, we vary the number of presets and compute the resulting system efficiency. To discriminate the error introduced by using transducer configuration presets from the errors introduced by tracking and motion prediction, we use again the ground-truth tracking and prediction components that know the underlying respiratory test motion. As the ultrasound shot duration is a critical parameter for this assessment, we also vary over this parameter. Figure 7 shows the resulting system efficiency. The reference is the temperature rise achieved with a real-time transducer (dashed plot). For subtle motion (scale factor *s*=0.5 a)) even 8 presets result in an efficiency of over 99%. For larger motions in b) and c) the efficiency with 8 preset quickly degrades. Using 32 or 64 presets is efficiency-wise close to using a real-time interface without presets. Temporal inaccuracies of the control loop execution threads can lead to cases where the preset-based approach is slightly more efficient than the real-time reference. To minimize this effect, the numerical experiment simulations were repeated 5 times and the results represent the mean efficiency.

### Estimated efficiency of the actual hardware FUS system

**Fig. 8:**
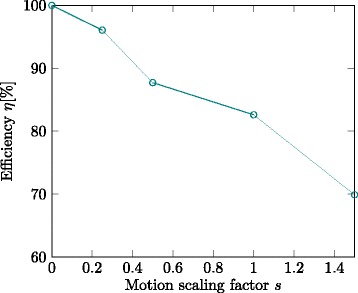
Estimated efficiency of the actual hardware FUS system based on the virtual system

Using the GE MR device, EPI (reduced field-of-view) images can be acquired with about 150 ms imaging duration and a delay of about 150 ms. The delay includes coil averaging and image reconstruction on the scanner, sending the data over the network and receiving it in our application. The InSightec CBS transducer is used in a preset-based approach that can handle 64 preset configurations. Switching between the presets is a matter of a few milliseconds. The system is equipped with Bayesian tracking, history-based motion prediction and a 50 ms shot duration. The presets are computed at the beginning of the monitoring session for a sonication based on the observed motion. Figure 8 gives the results as a plot of efficiency over motion scaling factor *s*. The overall efficiency of the virtual system with respect to temperature for normal motion is about 80%. For exaggerated motion (*s*=1.5) the efficiency goes down to about 70%. Less motion can be compensated with an efficiency greater than 80%.

## Discussion

In this paper, we describe the development of a novel FUS treatment system capable of performing motion-compensated application of FUS. Motion-compensation is achieved by deriving motion information from MR monitoring images and computing motion predictions to compensate delays in the processing loop. The system is developed with the goal of clinical use. A major problem is that developing a whole treatment system under such high quality regulations is a huge effort for research groups which normally is not compatible with short research projects of a few years. Usually, research groups are furthermore not trained and certified for the high quality standards necessary for the application of the system to humans. This challenge requires a change of the development process in the research context as we propose later.

To reduce the efforts necessary for testing and particularly real-world experimental validation, we here propose a simulation-based hardware emulation to test and analyze the efficiency of the system. The emulation internally uses a full numerical simulation of FUS during respiratory motion. Using this virtual system, choices of system parameters and algorithms can be determined with less effort. The actual real-world testing is thereby minimized to performing tests on the system resulting from the in-silico design stage.

In this work, we focus our attention to the motion compensation of the system. Firstly, we compute the limits of motion compensation for different choices of system parameter values for image duration and ultrasound shot duration. Even exaggerated motion (*s*=1.5) can theoretically be compensated in the case of an ultrasound shot duration of 300 ms with an efficiency above 90%. However, it would require a perfect temporal prediction which in practice is clearly not possible. To see how good the actual predictors can forecast the motion, we first individually analyze their influence on the system efficiency. This testing is done in combination with perfectly accurate tracking information. Afterwards, also the influence of motion tracking and also the transducer types is quantified. The motion tracking reduces the efficiency on average by only 4.9%. The effect of using a preset-based transducer instead of a real-time transducer is also analyzed. For the sole task of motion compensation, 32 presets should be enough to compensate even exaggerated motion. However, for the additional task of trans-costal delivery we assume that a larger number of presets is required. The temporal motion prediction is the most influential component. The history-based motion prediction is found to perform better than a linear-extrapolation-based predictor. We, however, see potential for improving the temporal predictions. A spatio-temporal motion model for liver motion [15], which currently is being integrated, is expected to improve the predictions. The model includes a temporal prediction method extending [18].

Finally, a virtual model of the actual hardware system with the same specifications (150 ms monitoring imaging delay, 150 ms image duration, 64-preset transducer) is tested and found to be capable of motion compensation of normal respiratory motion with an efficiency above 80%. Exaggerated motion (*s*=1.5) can be compensated with an efficiency of about 70%.

Potential for improving the system efficiency lies also in decreasing the overall computational delays in the system including computational delays introduced by the motion tracking, the motion predictors, and also imaging delays and update rates. Furthermore, we see potential in improving the temporal motion predictions by the combination of different tracking sources like external motion surrogates and ultrasound tracking.

## Conclusion

The reported efficiency values for the virtual model of our target hardware system are expected to be already suited for the clinical use of the motion compensation. We currently work on confirming the estimated efficiency of our system in real-world FUS experiments. Animal studies for further validation are in preparation. The long-term goal is to bring the technology to clinical use. Furthermore, we want to ease the translation of future research and build upon our current work in the future. The system might be a candidate for a platform for research developments in the FUS context. Research partners could contribute individual component implementations that are used in the system in a plugin fashion. A further long-term goal is to implement a challenge-style evaluation framework to automatically evaluate and compare the plugins using the simulation-based emulation. This approach could lead to more research results being transferred to clinical practice.
